# Diagnosis and Risk Prediction of Dilated Cardiomyopathy in the Era of Big Data and Genomics

**DOI:** 10.3390/jcm10050921

**Published:** 2021-02-26

**Authors:** Arjan Sammani, Annette F. Baas, Folkert W. Asselbergs, Anneline S. J. M. te Riele

**Affiliations:** 1 Department of Cardiology, Division Heart & Lungs, University Medical Center Utrecht, Utrecht University, 3582 CX Utrecht, The Netherlands; A.zabihisammani@umcutrecht.nl (A.S.); F.W.Asselbergs@umcutrecht.nl (F.W.A.); 2 Department of Genetics, Division Laboratories, Pharmacy and Biomedical Genetics, University Medical Centre Utrecht, University of Utrecht, 3582 CX Utrecht, The Netherlands; a.f.baas@umcutrecht.nl; 3 Institute of Cardiovascular Science, Faculty of Population Health Sciences, University College London, London WC1E 6BT, UK; 4 Health Data Research UK and Institute of Health Informatics, University College London, London WC1E 6BT, UK

**Keywords:** dilated cardiomyopathy, diagnosis, prognosis, big data, artificial intelligence, deep learning, genetic

## Abstract

Dilated cardiomyopathy (DCM) is a leading cause of heart failure and life-threatening ventricular arrhythmias (LTVA). Work-up and risk stratification of DCM is clinically challenging, as there is great heterogeneity in phenotype and genotype. Throughout the last decade, improved genetic testing of patients has identified genotype–phenotype associations and enhanced evaluation of at-risk relatives leading to better patient prognosis. The field is now ripe to explore opportunities to improve personalised risk assessments. Multivariable risk models presented as “risk calculators” can incorporate a multitude of clinical variables and predict outcome (such as heart failure hospitalisations or LTVA). In addition, genetic risk scores derived from genome/exome-wide association studies can estimate an individual’s lifetime genetic risk of developing DCM. The use of clinically granular investigations, such as late gadolinium enhancement on cardiac magnetic resonance imaging, is warranted in order to increase predictive performance. To this end, constructing big data infrastructures improves accessibility of data by using electronic health records, existing research databases, and disease registries. By applying methods such as machine and deep learning, we can model complex interactions, identify new phenotype clusters, and perform prognostic modelling. This review aims to provide an overview of the evolution of DCM definitions as well as its clinical work-up and considerations in the era of genomics. In addition, we present exciting examples in the field of big data infrastructures, personalised prognostic assessment, and artificial intelligence.

## 1. Introduction

Dilated cardiomyopathy (DCM) is defined as left ventricular (LV) dilation and systolic impairment in the absence of coronary artery disease or abnormal loading conditions. Even though robust data on the epidemiology of DCM are lacking, estimates suggest a disease prevalence of 1:125–250 in adults [1,2]. The disease is a clinically heterogeneous disease with large variability in sex, age of disease onset, and rate of progression, which is likely explained by the complex interplay between genetic susceptibility and environmental factors [3,4,5,6]. DCM is a leading cause of heart failure, and patients have a significant risk of life-threatening ventricular arrhythmias (LTVA) [7,8]. In the previous decades, considerable progress has been made in our understanding of the diagnosis, aetiology, and prognosis of DCM. For example, non-invasive tissue characterisation by cardiac magnetic resonance (CMR) has allowed for identification of fibrosis as a proarrhythmic marker, and the identification of disease-causing genetic variants enables early diagnosis and cascade screening of family members at risk of developing disease [9,10,11,12]. Clinical genetic testing is also important for prognosis, with specific genotypes (such as variants in Phospholamban (*PLN)* and Lamin A/C (*LMNA*)) being particularly prone to developing LTVA [8,13].

The large clinical heterogeneity of DCM results in many challenges in disease management. For instance, it is unclear if and to what extent relatives with pathogenic variants will develop DCM [5]. Regular surveillance and preventative treatment are therefore employed in at-risk individuals, but their effectiveness remains to be elucidated. In addition, prognosis of DCM is difficult to predict, as exemplified by the fact that the majority of DCM patients who receive an implantable cardiac defibrillator (ICD) do not receive appropriate interventions during follow-up. This emphasises the need for better risk prediction models [14].

New opportunities now lie in big data-minded clinical research. Electronic health records accompanied by big data infrastructures can improve accessibility of data. Newer methods such as machine and deep learning may improve clustering of patients that have similar characteristics and improve prognostic predictions in DCM. Rightfully so, the combination of big data and artificial intelligence (AI) has an increasing impact on the field of medicine [15,16,17,18,19]. This manuscript aims to provide an overview of DCM diagnosis and prognosis in the era of genomics and discusses exciting opportunities in the field of big data research and AI in DCM. Since this requires a better understanding of the chronology of DCM definitions, the secondary aim of this review is to provide a historic overview of DCM definitions pertinent to current clinical practice.

## 2. Defining DCM

### 2.1. Historic Overview of DCM Definitions

DCM constitutes an anatomic description of abnormal LV morphology and function in the absence of common pathophysiologic conditions (i.e., coronary artery disease or abnormal loading conditions). As such, it may be a final common pathway to many disease entities where outcome is strongly influenced by aetiology [7]. One of its first descriptions may be found in a case series by William Evans in 1948, describing “Familial Cardiomegaly” after excluding valvular, hypertensive, and congenital heart disease as causes of cardiac enlargement [20]. An autopsy in a subsequent family of two young sisters with “idiopathic cardiomegaly” also revealed dilatation of the LV, but the distinction between hypertrophic and dilated phenotypes was not yet as distinct as it is nowadays [21]. 

**Figure 1:**
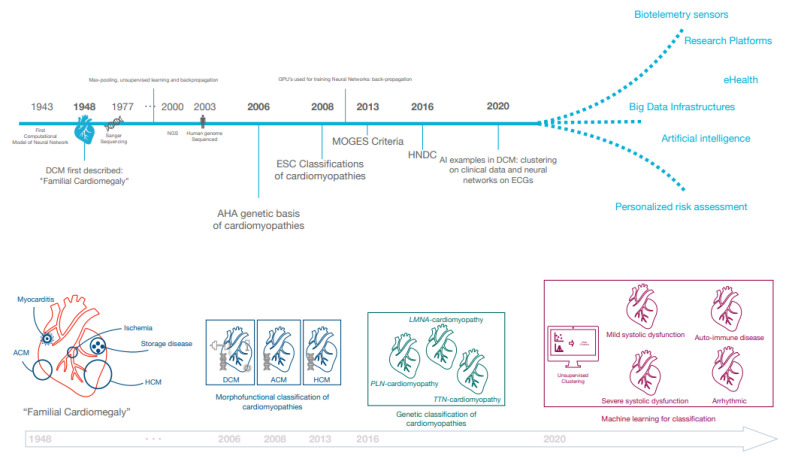
Historical milestones in the classification of dilated cardiomyopathy (DCM). A non-exhaustive list of historical milestones and future prospects are summarised. Additionally, the nomenclature from “familial cardiomegaly” to more specified disease is illustrated. Abbreviations: AHA (American Heart Association), ACM (arrhythmogenic cardiomyopathy), DCM (dilated cardiomyopathy), ESC (European Society of Cardiology), HCM (hypertrophic cardiomyopathy).

In the last decades, both European and American professional societies have proposed classifications of cardiomyopathic disorders (Figure 1). In 2006, the American Heart Association (AHA) published a seminal document describing the genetic basis of cardiomyopathies [22]. Subsequently in 2008, the European Society of Cardiology (ESC) emphasised that morphofunctional phenotype is the basis for cardiomyopathy classification and recognised extra-cardiac manifestations such as skeletal myopathy in cardiomyopathy patients [23]. The MOGE(S) classification was next proposed in 2013, which subclassified each of the cardiomyopathies into genetic forms and emphasised the necessity to further subdivide the DCM phenotype as it may affect prognosis and treatment [24]. While these position documents have greatly influenced our understanding of the phenotypic heterogeneity of DCM, the existing definitions remained limited in the case of intermediate phenotypes, such as in carriers of pathogenic genetic variants who may have incomplete disease expression. Similarly, LV systolic dysfunction or dilatation can be very mild or even absent in some acquired diseases, such as myocarditis. For these reasons, the ESC Working Group on Myocardial and Pericardial Disease proposed a revised definition including “hypokinetic non-dilated cardiomyopathy” (HNDC) as a marker of early or preclinical DCM in 2016 [5]. As per this framework, DCM (and HNDC) may be caused by genetic (±30%) and non-genetic (±70%) causes, of which the latter includes toxic substances (medication (antineoplastic, psychiatric antiretroviral), alcohol, cocaine, amphetamines, ecstasy, iron overload), nutritional deficiency, endocrinologic causes, tachycardiomyopathy, peri-partum cardiomyopathy, infection, and auto-immune disorders [3,5,7].

### 2.2. Diagnosis of DCM and Differential Diagnostic Considerations

DCM is considered in the presence of (1) left ventricular dilatation (indexed left ventricular end-diastolic diameter (LVEDd) >117% for age and sex, or the LV end-diastolic volume (LVEDV) ≥2 standard deviations from normal according to normograms), and (2) left ventricular systolic dysfunction (LV ejection fraction (LVEF) <45% and/or LV fractional shortening <25%). Normograms for echocardiographic volumes and diameters are available for adults and children and can be calculated using web-based calculators (e.g., www.parameterz.com, accessed on 15 February 2021). A full diagnostic work-up for DCM typically includes a focused history, laboratory evaluation, electrocardiography (ECG), Holter monitoring, echocardiography, CMR (with late gadolinium enhancement (LGE)), and genetic testing (Supplementary Table S1). In addition, differential diagnoses should be ruled out (e.g., ischemia detection to exclude coronary artery disease) [7].

Given that LV dilatation and dysfunction are the final common pathways in many heart diseases, other cardiomyopathies (arrhythmogenic cardiomyopathy (ACM), hypertrophic cardiomyopathy (HCM), non-compaction cardiomyopathy (NCCM), and restrictive cardiomyopathy (RCM)) may mimic the DCM phenotype [25]. For instance, end-stage HCM may show overlapping clinical characteristics (LV dilatation and reduced LVEF), and ACM may present with a biventricular or left-dominant phenotype [2,9,26,27,28]. Additionally, Chagas disease, which is caused by a parasite (*Trypanosoma cruzi*), may lead to cardiomyopathy in 30–40% of affected individuals [29]. To distinguish DCM from common differential diagnostic considerations, research has proven that CMR is very useful in recent years as it provides a good visualisation of not only the LV but also the right ventricular (RV) myocardium [9]. In addition, LGE patterns on CMR may assist in determining the aetiology—while not mutually exclusive, LV midwall LGE may be seen in genetic forms of DCM or myocarditis, whereas subepicardial LGE may be caused by myocarditis, sarcoidosis, or chemotherapy [30]. Myocarditis may also cause DCM by a complex interplay of inflammation and (auto)-immunologic response [25]. A definite diagnosis of myocarditis, however, cannot be made without endomyocardial biopsy and should focus on immuno-histochemistry (“Dallas criteria”) and detection of DNA or RNA of the infectious agent [31,32].

## 3. Classification of DCM in the Era of Genomics

Genetic testing has greatly increased our understanding of the aetiology of DCM and has led to the identification of individuals at risk of developing disease [10,11,12]. In recent years, next-generation sequencing (NGS) has tremendously accelerated genetic testing in DCM given its low cost, flexibility, short turnaround time, and genome-wide coverage. NGS gene panels and whole-genome sequencing can be used to identify pathogenic point variants, small insertions and deletions, or large structural copy number variations [2,33,34]. As a result, NGS now is the clinical gold standard for genetic evaluation of DCM.

### 3.1. Genetic Variants in DCM

**Figure 2:**
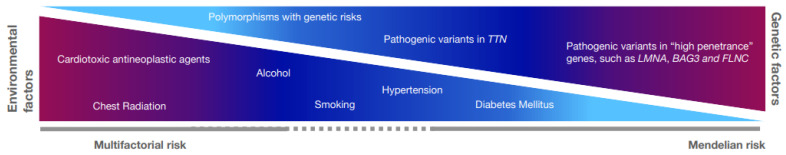
Schematic overview of genetic susceptibility and environmental factors affecting dilated cardiomyopathy. This schematic overview illustrates on the right a more mendelian risk profile with pathogenic variants in “high penetrance” genes versus a more multifactorial risk profile on the left. Importantly, the truth may be a combination of both, e.g., Titin (*TTN*) variants in patients with dilated cardiomyopathy with alcohol abusus. Polygenic risks may also affect disease in pathogenic variants in “high penetrance” genes.

Most common pathogenic variants in DCM patients are identified in genes encoding sarcomere proteins (e.g., Titin (*TTN*) and Myosin Heavy Chain 7 (*MYH7*)); Z-disk components (e.g., Filamin-C (*FLNC*) and BLC2 Associated Athanogene 3 (*BAG3*)); and in the *LMNA* gene, encoding a structural protein of the nuclear envelope [10,11,12]. Variant interpretation, however, requires due diligence, as pathogenic variants may have been overreported in the past and variant reclassification has direct implications on patients and at-risk relatives. To this end, the ClinGen consortium has been re-evaluating reported pathogenic variants to generate international consensus on variant interpretation [35,36]. However, even with first-rate variant curation, genetic variation only explains up to 40% of DCM cases. In patients without a family history of DCM (i.e., sporadic DCM), this yield can decrease to 10%, suggesting a bigger role for non-genetic causes including cardiotoxic medication (anthracycline), alcohol, and inflammation (Figure 2) [3,4,5,37,38,39].

Truncating variants in *TTN* (*TTNtv)* are the most commonly known genetic cause of DCM. Because of its size (17,106 base pairs), *TTN* diagnostic testing has become available only after the introduction of NGS. These disease-causing variants are mostly located in the A-band region. *TTN* nonsense, frameshift, splicing, and copy-number variants likely cause haplo-insufficiency and are significantly more frequent among subjects with DCM (20%) than in the general population (up to 0.5%) [40,41,42]. Furthermore, variants in *TTN* are overrepresented in patients with external triggers for DCM, including alcohol, chemotherapy, and pregnancy, illustrating the interplay between genetic predisposition and environmental factors and supporting the multifactorial disease model for DCM [4,6,40,41,43]. As such, genetic testing for TTN by NGS can be useful in all DCM patients to allow for earlier diagnosis and therapeutic intervention. Interestingly, most patients with DCM caused by *TTNtv* have a relatively mild disease course and respond well to treatment, although some studies have reported a higher burden of LTVA, irrespective of LVEF in these patients [44,45,46,47,48]. The significance of *TTNtv*, however, remains uncertain in unaffected relatives and individuals tested for a non-DCM disease. Long-term outcome studies in unaffected carriers are necessary to determine appropriate management recommendations in these subjects [41,47,49]. 

### 3.2. Genotype–Phenotype Associations in DCM

In recent years, we have come to appreciate genotype–phenotype associations within the spectrum of DCM [13]. For instance, microvoltages and frequent ventricular extrasystoles on ECG and Holter monitoring are often seen in carriers of *PLN* pathogenic variants, while in *LMNA*, variant carriers a low p-wave amplitude, and prolongation of the PR interval with narrow QRS complexes are typically observed [13,50,51]. The combination of distinct phenotypic features together with their genetic aetiology resulted a new nomenclature of these clinical entities, such as “*PLN-*cardiomyopathy” and “*LMNA-*cardiomyopathy” [52,53,54].

**Figure 3:**
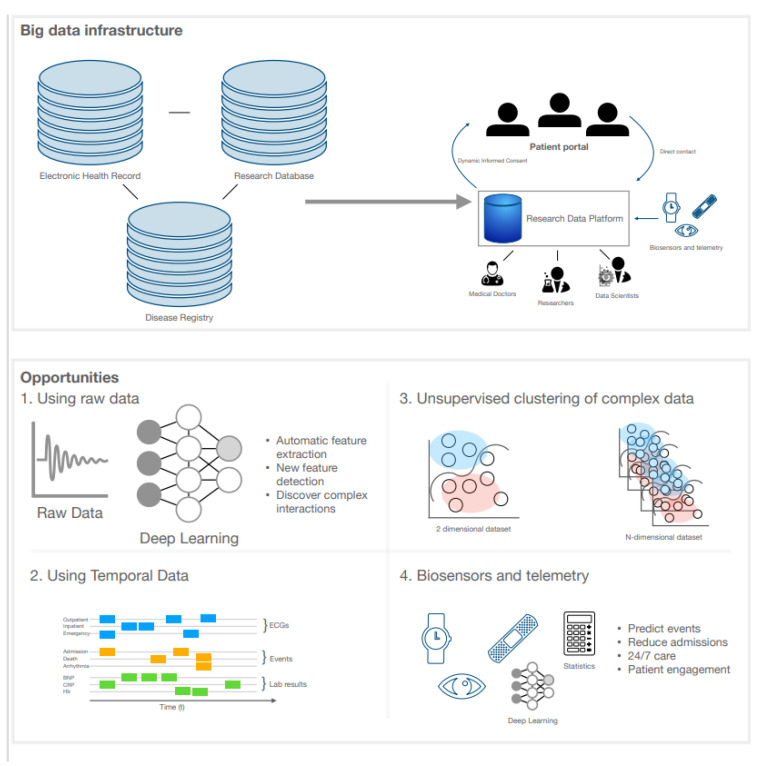
Big data infrastructure and opportunities using artificial intelligence, eHealth, and wearables in the management of DCM. An interoperable big data infrastructure is visualised with the possibility of research data platforms as exemplified in the text. The field is now ripe with opportunities to be explored by the eager-minded using research data platforms and using raw uninterpreted data, temporal datasets, unsupervised learning of more dimensional datasets, and the embedding of biosensors and telemetry. Abbreviations: ECG (electrocardiogram).

Genotype–phenotype association studies not only described diagnostic features, but also focussed on clinical outcome in DCM patients. As a result, we now know that pathogenic variants in *BAG3* are characterised by a significant risk of progressive heart failure [55]. In addition, *LMNA*, *PLN*, *TNNT2*, and *MYH7* variants are frequently identified in patients who undergo heart transplantation. Likewise, we now know that patients with pathogenic variants in *FLNC*, *LMNA*, *PLN*, *RBM20*, *SCN5A*, and *TTN*, and desmosomal genes carry a higher burden of arrhythmia (Figure 3) [8,13,46]. In general, DCM patients with pathogenic variants more often have unfavourable outcome than patients without a pathogenic variant. This was already described in a study of 418 DCM index patients, among whom 61% of pathogenic variant carriers had a major adverse clinical event as compared to 22% of genetically unexplained cases [38]. However, the *TTN* gene was not evaluated in this cohort. It is possible that a majority of the variant negative patients from this study may have harboured *TTNtv* [47]. We encourage readers to view the previously published gene lists of DCM-associated pathogenic variants and their genotype–phenotype associations [2,13,33,56,57,58].

### 3.3. Genome-Wide Association Studies and Genetic Risk Scores in DCM

An explanation for the limited diagnostic yield of monogenetic causes and incomplete penetrance of DCM-associated variants is a common genetic variation [59]. To understand the relationship between these common genetic variants and DCM, researchers conducted several case-control genome-wide association studies (GWASs) and one exome-wide association study (EWAS) [60,61,62,63]. The three GWASs identified several loci including the following genes: *HSPB7*, *BAG3*, *HCG22*, *SLC6A6*, and *SMARCB1* [60,61,63]. The EWAS reported eight loci independently associated with sporadic DCM, five of which included genes that harbour rare DCM causing variants (*TTN*, *ALPK3*, *BAG3*, *FLNC*, and *FHOD3)* [62]. Additionally, a recent GWAS conducted in individuals from the UK Biobank investigated the role of genetic associations in CMR-derived LV measurements. They identified 45 previously unreported common genetic loci that were associated with cardiac function and dimensions in individuals without cardiovascular disease [59]. The results of these studies indicate that common genetic variation plays an important role in DCM development and progression.

To this end, genetic risk scores may be used to estimate an individual’s lifetime genetic risk of a disease, which can be a useful tool to discriminate that subjects require more frequent surveillance [5,64]. Polygenic risk scores were constructed on the basis of the identified loci that were linked to DCM, one of which comprised 28 single nucleotide polymorphisms that was able to predict DCM with a hazard ratio (HR) of 1.58 per standard deviation increase in the risk score. Moreover, LV end systolic volume and LVEF of *TTNtv* carriers were also shown to be influenced by this polygenic risk score [59]. This study, however, also underscored the particularly challenging clinical validation and implementation of polygenic risk scores: (1) the scores were mostly developed in patients with European/Western ancestry, and (2) interpretation was based on the distribution of risk, which may limit information on an individual’s lifetime risk [65]. As previously mentioned, whether or not a carrier of a pathogenic variant develops DCM will also depend on the influence of environmental and cardiotoxic factors, further complicating risk predictions (Figure 2). Future research in large, well-phenotyped cohorts of pathogenic variants is required to define the utility of these genetic risk scores for individual prognosis. 

## 4. Prognosis of DCM

### 4.1. Heart Failure and Cardiac Transplantation

Historically, DCM had a 1-year mortality of ±30% and 5-year mortality of up to ±50% [66]. Because of therapeutic advancements, however, mortality rates have been decreasing, leading to 5-year mortality rates of 20% nowadays [66,67]. In general, patients with DCM are at risk of frequent hospitalisation and overt heart failure, for which left ventricular assist devices (LVAD) and orthotopic heart transplantation (HTx) are the effective last resort treatments [68]. Independent predictors of progressive heart failure include low LVEF, RV dilatation, global segmental wall motion abnormalities, high New York Heart Association (NYHA) class, older age, male sex, the presence of conduction disorders, and LGE on CMR (i.e., fibrosis) [66,69]. Other predictors include reduced exercise capacity, low systolic blood pressure, and low haemoglobin [70].

Several clinical prediction models have been constructed for heart failure in the general cardiology population in order to facilitate prognostic assessments. The Seattle Heart Failure Model (SHFM) [71], MAGGIC risk score [72], and the Barcelona bio-HF calculator [73] are three models with comparable risk prediction performance [74]. The performance of these models in DCM may, however, be suboptimal because their derivation also included patients with ischemic aetiology who are known to have a higher mortality risk than DCM patients (3-year mortality between 24–40%) [37,74]. Indeed, a recent comparison of these prediction models in an external DCM cohort produced an area under the curve (AUC) of ≥0.6, with the more sophisticated risk models (BCN Bio-HF and SHFM) yielding the highest accuracies [71,73,74]. Interestingly, the risk models typically overestimated mortality risk in DCM patients. This may be caused by a difference in age (DCM patients tend to be younger (±15 years) than other heart failure aetiologies), which is one of the strongest variables affecting mortality [37,74]. Moreover, there is a distinct subgroup of DCM patients who experience LV function recovery and have a subsequent mild clinical course during follow-up [75]. These patients typically have higher LV contractile reserve and are more often women, whereas the presence of LGE on CMR in patients with DCM often represents an ominous marker [76,77]. Larger prospective studies are warranted for two purposes: (1) to discriminate patients who may recover and better understand their pathophysiologic substrates which may impact new treatment strategies, and (2) to develop a risk stratification tool dedicated for DCM patients that also incorporates variables reflecting LV recovery [74,77,78]. 

### 4.2. Life-Threatening Ventricular Arrhythmias

Patients with DCM have a ±4.5% annual risk of LTVA and therefore may benefit from ICD implantation [8,79]. Current guidelines provide a Class IB recommendation for ICD implantation in symptomatic (NYHA ≥ II) DCM patients with an LVEF ≤35%, despite ≥3 months of optimal pharmacological therapy [80]. However, not all patients with a low LVEF derive benefit from ICD implantation, and improved selection of these patients is warranted [8]. It seems obvious that those with prior sustained ventricular arrhythmias (i.e., secondary prevention) should receive an ICD, whereas recommendations for primary prevention cases are less straightforward as illustrated by the negative DANISH trial [81]. 

Several studies have evaluated risk factors for LTVA in DCM, which include reduced LVEF; LV dilatation; LGE on CMR; prior (non)sustained ventricular arrhythmia; and pathogenic variants in *PLN*, *LMNA*, *FLNC*, and *TTNtv* [8,48,82,83]. A recently published post hoc analysis of the MADIT trials in heart failure patients (including those with ischemic aetiology) confirmed that low LVEF (≤25%), male sex, prior non-sustained ventricular tachycardia, atrial arrhythmia, and myocardial infarction are potent predictors of LTVA [84]. An important consideration would be the confounding effect of cardiac resynchronisation therapy defibrillator (CRT-D) devices as these devices influence the risk of LTVA (including appropriate device therapy) in two ways: (1) they may decrease the risk of arrhythmia (improved LVEF with LV remodelling) and (2) they increase the detection chance ventricular arrhythmias, including self-terminating and possibly non-life threatening ones [85,86]. Moreover, newer techniques such as LGE on CMR and genetic sequencing are gaining ground in the work-up of DCM, which should be incorporated in future risk scores for DCM as they may improve ICD candidate selection even further [8].

## 5. Big Data Research Opportunities and Artificial Intelligence in DCM

Big health records are beginning to adjust the nature of research and clinical medicine. They contain data that can improve our understanding of disease causation, classification, and prognosis [87]. The use of electronic health records (EHR) has improved accessibility of these data and methods such as machine and deep learning can model complex interactions by finding new phenotype clusters, classifying diseases, or predicting prognosis (Figure 3) [17]. 

### 5.1. Big Data Infrastructure

Big data are usually described as data that are high in volume (e.g., by number of patients) and high in complexity (e.g., by temporality or number of variables) [87]. The phenotypic data in an EHR system complies with this definition, as it may include detailed data on laboratory values, investigations, raw imaging and ECG data, device data, (unstructured) text, and questionnaires in a large number of patients [15]. As each patient has a multitude of time-stamped events and data points that are performed upon discretion of the treating physician, these data are high dimensional, sparse (with varying intervals between data points), irregular, and temporal. Furthermore, data may be biased because of administrative or financial interests or because of a highly variable yet meaningful missingness [88]. 

These challenges may be overcome by several methods. Phenotype-classifying algorithms may be able to cope with conflicting or missing data by combining multiple data sources and integrating information on treatment and comorbidities to infer diagnoses, as shown by a case study in atrial fibrillation [89]. Furthermore, clinically repetitive or administrative tasks can be automated [90]—for instance, EchoNet is a deep learning network that can accurately extract LV volume and function, and other algorithms have been deployed for automatic CMR multi-structure segmentation [91,92]. Registration of diagnoses can also be automated, for example by interpreting clinical discharge letters and extracting diagnoses using deep learning [93,94]. Of note, these pipelines do not always need to rely on complex algorithms. Boolean retrieval methods or regular expressions are two examples of simpler algorithms that are also capable of extracting data from medical text [95]. These pipelines help creating big data research infrastructures by extracting previously messy data and structuring them for clinical research and patient care. Big data research infrastructures can also be a combination of conventional cohorts, e.g., EHR in population settings, disease registries, and trial data. BigData@Heart is an example that combines these different research and population datasets to gather real-world evidence [96]. As EHR systems are continued to be used as research data platforms, interoperability is important for continuous collaborations and validation of algorithms. When validated, these algorithms can then be entered into prospective clinical trials and implemented in clinical practice given that their big data infrastructure is already in place. Integrated data repositories and consensus-based approaches for data modelling and a variety of data models have been developed to provide standardisation, the latest being the OMOP Common Data Model and HL7 FHIR (Fast Healthcare Interoperability Resources) [97].

### 5.2. Clinical Uses of Artificial Intelligence in Cardiomyopathy

Deep neural networks can discover complex patterns in data and be trained (and tested) to classify diseases. For example, a recent deep neural network accurately classified pathogenic *PLN* variants on the basis of 12-lead ECGs in patients with cardiomyopathy [98]. The network showed excellent discriminatory performance and visualised both established ECG features (low QRS voltage and T-wave inversions) and a novel disease-specific ECG feature (increased PR duration) [98]. Another deep neural network was able to accurately triage ECG into four categories (normal, abnormal not acute, subacute, and acute) [99]. Unsupervised clustering algorithms may also be able to help our understanding of phenotypic heterogeneity in DCM, as they can identify pathophysiologically similar individuals who may respond in a uniform and predictable way to treatment [100]. Indeed, a recent study identified four different DCM phenotype subgroups (“phenogroups”) using an unsupervised hierarchical clustering algorithm, which was validated in two external registries: the Italian Trieste Registry and a cohort from Madrid [4,101,102]. The four identified phenogroups were summarised as (i) mild systolic dysfunction, (ii) auto-immune disease, (iii) arrhythmic, and (iv) severe systolic dysfunction. The latter three groups had comparable and relatively unfavourable outcome compared to the first phenogroup [101]. Whether these subgroups can be used to guide clinical decision-making remains to be investigated [5]. Since the prevalence of subtle systolic and diastolic dysfunction is present in genotype-positive phenotype-negative DCM relatives, using machine and deep learning for early detection of disease may be an important next step [103]. It is, however, essential to appreciate that implementation of these techniques is a multi-disciplinary effort with data scientists (who make the models), “hybrid physicians” (who speak the “AI language”), and IT specialists (who provide the infrastructure) [87].

### 5.3. eHealth and Wearables in DCM Management

Wearables and smartphones are embedded with (and connected to) many sensors that play a leading role in healthcare, ranging from accelerometers, temperature/heart rate detectors, and ECGs. To engage clinicians to use these data, platforms have been created to facilitate data storage and connectivity with these wearables, such as RADAR-base (an open-source mobile health platform) or the Harvard Forhealth application [104,105]. These applications allow for dynamic informed consents with patients and direct connection to researchers, while simultaneously providing the infrastructure for the assessment of telemonitoring devices. These initiatives mark the beginning of a paradigm shift from “one-size-fits-all” to personalised care supported by AI [106]. 

Indeed, the last year has brought an exponential increase in studies using various forms of eHealth, which are now moving from research to implementation [107]. A recent meta-analysis estimated that telemonitoring systems reduce all-cause mortality in heart failure patients by ±20%, with optimal results if ≥3 simple biologic parameters (body weight, blood pressure, or ECG) are measured [108]. For heart failure hospitalisations in patients with CRT devices, however, older data indicate that more complex diagnostic indices are necessary: decreased intrathoracic impedance, low patient activity, and low heart rate variability (HRV) [109]. More specifically, a HRV of <50 ms (standard deviation of the 5-min atrial–atrial intervals) was associated with high mortality and hospitalisation risk [110]. Non-invasive disposable patches were also shown to accurately provide early detection of impending rehospitalisation when combined with a machine learning algorithm [111]. Whether these innovative approaches can mitigate rehospitalisation (if acted upon) remains to be investigated. In addition, the benefit of wearables for arrhythmia detection or pre-clinical disease detection (e.g., by ECGs) in at-risk individuals needs to be evaluated in large prospective studies [112].

## 6. Conclusions

Clinical management of DCM patients is challenging given the large heterogeneity in disease phenotype, genetic background, and progression of disease. Interoperable big data infrastructures comprising EHRs, registries, and other patient databases can now be used with new techniques, such as deep and machine learning, in order to identify phenotype clusters, assess new features that classify DCM phenotypes, and predict disease outcome and validate them across different international cohorts. As technology advances, eHealth using wearables provide exciting new opportunities to personalise care and move towards patient-tailored predictive and preventive medicine. 

## Supplementary Materials

The following are available online at https://www.mdpi.com/2077-0383/10/5/921/s1, title, Table S1: Non-exhaustive list of clinical modalities that may aid in differential diagnosis of DCM
