# Supplementary material for: Diagnosis and Risk Prediction of Dilated Cardiomyopathy in the Era of Big Data and Genomics
Source: J Clin Med. 2021 Feb 26;10(5):921. doi: 10.3390/jcm10050921 (PMC7956169; doi:10.3390/jcm10050921)
Supplement: Supplementary file 1 [file jcm-10-00921-s001.pdf]

Journal of
Clinical Medicine

MDPI

Table 1. Non-exhaustive list of clinical modalities that may aid in differential diagnosis of DCM.

Modality	DCM	Ischemic HF	ACM	HCM	NCCM	RCM
**History**	Possible positive family history, young age	Atherosclerosis, older age	Possible positive family history, young age, syncopes or palpitations	Possible positive family history, young age	Possible positive family history, young age	Possible positive family history, young age
**ECG**	Low QRS voltage, conduction disorders, left axis deviation, t-wave abnormalities*	Pathological Q waves, ST segment abnormalities, conduction disorders	Inverted T-waves, late potentials, epsilon wave, and/or terminal activation delay typically preceding structural changes	High QRS voltages indicative of LVH and abnormal repolarisation	ECG characteristic of LVH and abnormal repolarisation	Low QRS voltage, conduction disturbances, pseudo-infarction Q-waves
**Echocardiography**	Dilated LV and/or systolic impairment	Dilated ventricle with systolic impairment, typically by coronary perfusion region	RV dilatation or dysfunction, RV dyskinesia or aneurysm. Abnormal LV function in absence of RV dysfunction is rare (left-dominant ACM).	LVH (≥13 mm), diastolic dysfunction, obstructed LV outflow tract, (supra)normal systolic function	Ventricular hypertrophy with compacted and non-compacted (trabeculae) myocardium	Not dilated ventricular with normal or increased wall thickness, restrictive filling pattern (Doppler) and atrial dilation
**CMR**	Dilated LV and systolic impairment, possible hypokinetic wall motion abnormalities, delayed enhancement (mid-wall)	Possible dilated LV and systolic impairment, akinetic wall motion abnormalities, subendocardial delayed enhancement	Regional RV akinesia, dyskinesia or dysschronous RV contraction, biventricular fibrofatty replacement of LV or RV wall.	LVH, apical and lateral wall hypertrophy, obstructed LV outflow tract, septal delayed enhancement	Hypertrabeculation (noncompacted to compacted ratio of >2.3), trabeculated LV mass >20% of global LV mass	Normal sized ventricles, enlarged atria, tubular or indented ventricular cavities, extra-cardiac findings associated amyloidosis/ sarcoidosis
**Genetic Testing**	Pathogenic variants in sarcomeric, desmosomal and/or nucleair envelope proteins	Not indicated	Pathogenic variants in mostly desmosomal proteins	Pathogenic variants in mostly sarcomeric proteins	Pathogenic variants in mostly sarcomeric proteins	Dependent on aetiology of RCM
**CAG**	Normal	Abnormal (or normal in case of microvascular disease or coronary spasm)	Normal	Normal	Normal	Normal

* Genotype-phenotype associations may shed light on the aetiology of DCM. See text for examples. Abbreviations: ACM (arrhythmogenic cardiomyopathy), CMR (cardiac magnetic resonance imaging), CAG (coronary angiogram), DCM (dilated cardiomyopathy), ECG (electrocardiogram) HCM (hypertrophic cardiomyopathy), LV (left ventricle) LVH (left ventricular hypertrophy), RCM (restrictive cardiomyopathy), RV (right ventricle).
